# Supplementary material for: Diversity-triggered deterministic bacterial assembly constrains community functions
Source: Nat Commun. 2019 Aug 23;10:3833. doi: 10.1038/s41467-019-11787-5 (PMC6707308; doi:10.1038/s41467-019-11787-5)
Supplement: Supplementary file 1 — Supplementary Information [file 41467_2019_11787_MOESM1_ESM.pdf]

Supplementary Information

Diversity-triggered deterministic bacterial assembly constrains community functions

Authors: Weibing Xun[1, 2], Wei Li[1], Wu Xiong[1], Yi Ren[1], Yunpeng Liu[2], Youzhi Miao[1], Zhihui Xu[1], Nan Zhang[1], Qirong Shen[1*], Ruifu Zhang[1, 2*]

Affiliations:

[1]Jiangsu Provincial Key Lab of Solid Organic Waste Utilization, Jiangsu Collaborative Innovation Center of Solid Organic Wastes, Educational Ministry Engineering Center of Resource-saving fertilizers, Nanjing Agricultural University, Nanjing, 210095, China

[2]Key Laboratory of Microbial Resources Collection and Preservation, Ministry of Agriculture, Institute of Agricultural Resources and Regional Planning, Chinese Academy of Agricultural Sciences, Beijing 100081, P.R. China

***Corresponding authors**:

Ruifu Zhang & Qirong Shen, College of Resources & Environmental Sciences, Nanjing Agricultural University, 210095, Nanjing, China; Correspondence should be addressed to R.Z (email: rfzhang@njau.edu.cn) and Q.S (email: shenqirong@njau.edu.cn).

List of content:

Supplementary Figures: 2

Supplementary Tables: 5

Supplementary Note (R codes): 1

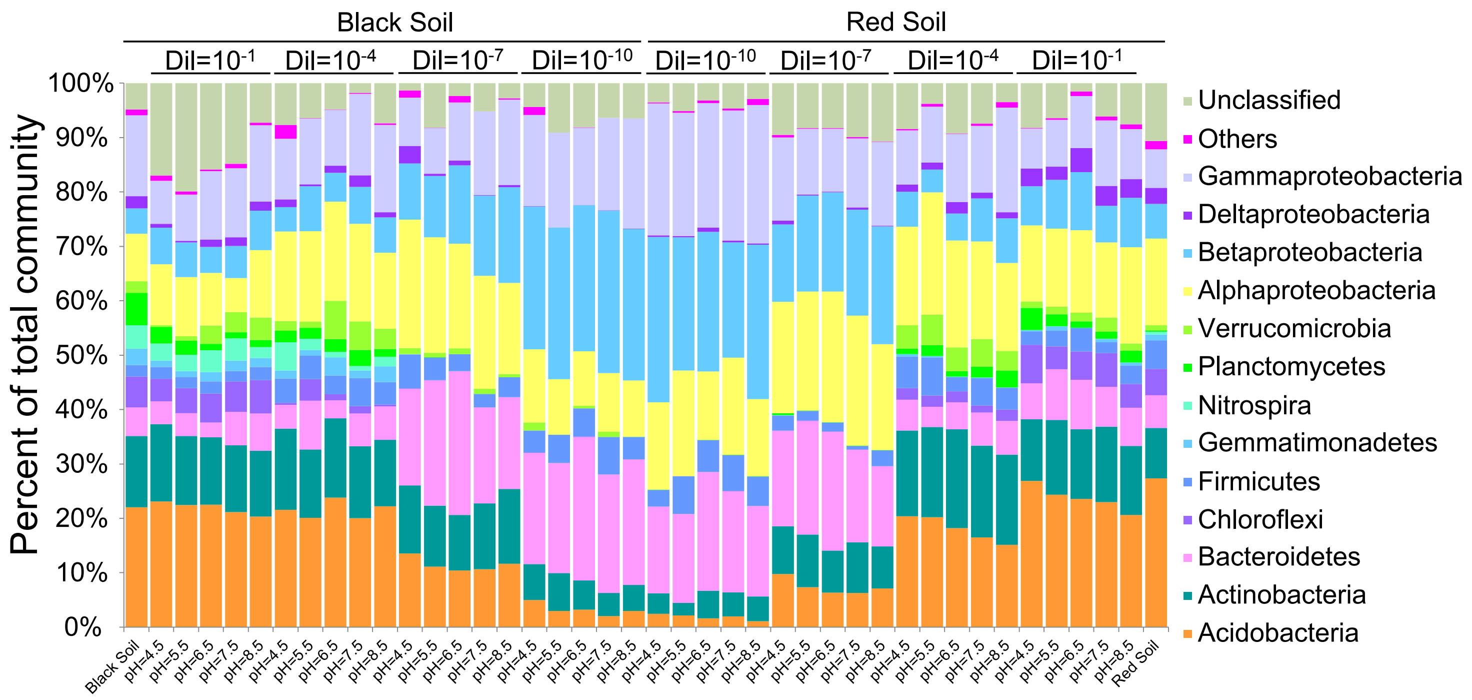

Supplementary Figure 1. Taxonomic distributions of re-assembled bacterial community at phylum level (class level for Proteobacteria) based on amplicon sequencing data. Dil indicates the dilution level.

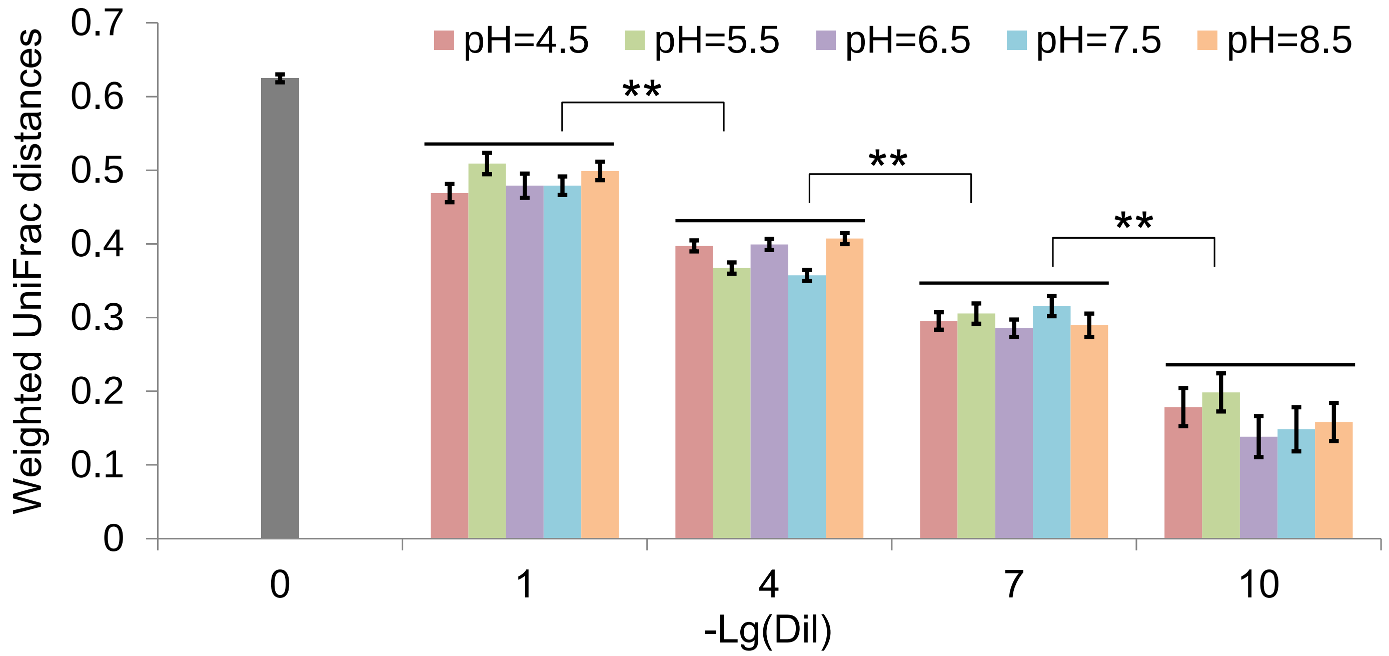

Supplementary Figure 2. Weighted Unifrac distances of pairwise bacterial communities between black soil and red soil at the same pH and dilution level. Dil indicates the dilution level. -Lg(Dil): Lg transformed dilution level. -Lg(Dil) = 0 represents the untreated soil. **, *P*-value < 0.001 based on t-tests. Error bars represent standard deviations (n = 144).

Supplementary Table 1. The number of unique genes and the increased and decreased genes which are significantly associated with dilution for each functional category.

Functional catergory	No. Unique Gene	Sig. Decreased	Sig. Increased
Glycolysis/Gluconeogenesis	151949	4326(2.85%)	66158(43.54%)
TCA cycle	75595	1495(1.98%)	31542(41.72%)
Pentose phosphate pathway	68622	3614(5.27%)	35498(51.73%)
Fructose & Mannose metabolism	79299	2841(3.58%)	40580(51.17%)
Galactose metabolism	42364	955(2.25%)	25481(60.15%)
Starch & Sucrose metabolism	72156	5857(8.12%)	38475(53.32%)
Pyruvate metabolism	101546	3029(2.98%)	51439(50.66%)
Butanoate metabolism	66624	1413(2.12%)	41273(61.95%)
Glyoxylate & Dicarboxylate metabolism	99822	2747(2.75%)	47616(47.70%)
Propanoate metabolism	61750	4682(7.58%)	21542(34.89%)
Amino acid metabolism	765657	281462(36.76%)	94560(12.35%)
Lipid metabolism	233079	70134(30.09%)	22453(9.63%)
Xenobiotics biodegradation/metabolism	98384	30943(31.45%)	1774(1.80%)
Terpenoids & Polyketides metabolism	120489	75468(62.63%)	2016(1.67%)
Methane metabolism	53827	34915(64.87%)	3240(6.02%)
Nitrogen metabolism	85115	39470(46.37%)	7844(9.22%)
Sulfur metabolism	38915	21308(54.76%)	1856(4.77%)

Percentages in brackets are the proportions of the increased or decreased genes to the number of unique genes for each functional category. The correlations were calculated using Spearman correlations. The significant associated genes were counted with the statistical r-value > 0.60 and P-value < 0.05, two-sided tests.

Supplementary Table 2. Indicator values obtained by indicator species analysis for functional genes which are strongly associated with dilution

KEGG_gene ID	KO_ID	KO_name	KO_definition	KO_EC	KO_class_1	KO_class_2	Enriched/Depleted	IndVal	p
msp:Mspyr1_23710	K01523	hisE	phosphoribosyl-ATP pyrophosphohydrolase	3.6.1.31	Amino Acid Metabolism	Histidine metabolism	Enriched	0.803	0.001
rpb:RPB_3640	K02610	paaB	ring-1,2-phenylacetyl-CoA epoxidase subunit PaaB	--	Amino Acid Metabolism	Phenylalanine metabolism	Depleted	0.653	0.002
rha:RHA1_ro01853	K00014	aroE	shikimate dehydrogenase	1.1.1.25	Amino Acid Metabolism	Phenylalanine, tyrosine and tryptophan biosynthesis	Depleted	0.539	0.001
bam:Bamb_4307	K03392	E4.1.1.45, ACMSD	aminocarboxymuconate-semialdehyde decarboxylase	4.1.1.45	Amino Acid Metabolism	Tryptophan metabolism	Depleted	0.697	0.002
rpb:RPB_1000	K00666	K00666	fatty-acyl-CoA synthase	6.2.1.-	Lipid Metabolism	Lipid biosynthesis proteins	Enriched	0.913	0.001
bgf:BC1003_0498	K00252	GCDH, gcdH	glutaryl-CoA dehydrogenase	1.3.99.7	Lipid Metabolism	Fatty acid metabolism	Enriched	0.503	0.004
syn:sll1441	K10257	FAD8, desB	omega-3 fatty acid desaturase (delta-15 desaturase)	1.14.19.-	Lipid Metabolism	Biosynthesis of unsaturated fatty acids	Depleted	0.642	0.002
aba:Acid345_2787	K01061	E3.1.1.45	carboxymethylenebutenolidase	3.1.1.45	Xenobiotics Biodegradation and Metabolism	Fluorobenzoate degradation	Depleted	0.644	0.002

Continued table									
bur:Bcep18194_C7122	K03379	E1.14.13.22	cyclohexanone monooxygenase	1.14.13.22	Xenobiotics Biodegradation and Metabolism	Caprolactam degradation	Depleted	0.757	0.001
bcj:BCAL2029	K03382	atzB	hydroxyatrazine ethylaminohydrolase	3.5.99.3	Xenobiotics Biodegradation and Metabolism	Atrazine degradation	Depleted	0.653	0.003
bra:BRADO4348	K08684	E1.14.13.25	methane monooxygenase	1.14.13.25	Methane metabolism	Methane metabolism	Depleted	0.665	0.003
rpc:RPC_0952	K02567	napA	periplasmic nitrate reductase NapA	1.7.99.4	Nitrogen metabolism	Nitrogen metabolism	Enriched	0.938	0.001
acm:AciX9_0125	K00372	E1.7.99.4C	nitrate reductase catalytic subunit	1.7.99.4	Nitrogen metabolism	Nitrogen metabolism	Depleted	0.698	0.001
azc:AZC_1039	K02591	nifK	nitrogenase molybdenum-iron protein beta chain	1.18.6.1	Nitrogen metabolism	Nitrogen metabolism	Depleted	0.771	0.001
bps:BPSS1243	K00362	nirB	nitrite reductase (NAD(P)H) large subunit	1.7.1.4	Nitrogen metabolism	Nitrogen metabolism	Depleted	0.812	0.001
ach:Achl_2819	K00390	cysH	phosphoadenosine phosphosulfate reductase	1.8.4.8	Sulfur metabolism	Sulfur metabolism	Depleted	0.946	0.002
pfv:Psefu_2275	K00381	cysI	sulfite reductase (NADPH) hemoprotein beta-component	1.8.1.2	Sulfur metabolism	Sulfur metabolism	Depleted	0.918	0.001

Enriched: the gene was enriched by dulution; Depleted: the gene was depleted by dilution.

Supplementary Table 3. The added amount of CaO and $FeSO_4$ to manipulate soil pH.

Soil pH	Black Soil (pH = 8.0)		Red Soil (pH = 5.3)	
	1M $FeSO_4$ (ml kg^{-1})	0.5M CaO (ml kg^{-1})	1M $FeSO_4$ (ml kg^{-1})	0.5M CaO (ml kg^{-1})
4.5	98.7	0	9.5	0
5.5	26.4	0	0	1.2
6.5	11.2	0	0	14.7
7.5	2.5	0	0	38.3
8.5	0	1.3	0	67.5

Supplementary Table 4. The concentrations of Fe, Ca and SO_4^{2-} of soil samples after incubation.

Soil Sample	Fe（g kg^{-1}）	Ca (g kg^{-1})	SO_4^{2-} (mmol kg^{-1})
Untreated Black Soil	16.1 (0.6**d**)	11.05 (0.7a**b**)	4.1 (0.06**e**)
Black Soil (pH=4.5)	24.7 (0.7**a**)	10.25 (0.4**b**)	21.5 (0.16**a**)
Black Soil (pH=5.5)	19.5 (0.2**b**)	9.26 (0.6**c**)	8.6 (0.08**b**)
Black Soil (pH=6.5)	17.6 (0.7**c**)	12.01 (0.5**a**)	4.6 (0.07**c**)
Black Soil (pH=7.5)	16.9 (1.0**cd**)	11.85 (1.2**a**)	4.4 (0.12**d**)
Black Soil (pH=8.5)	15.5 (0.8**d**)	12.19 (1.4**a**)	4.4 (0.19**d**)
Untreated Red Soil	31.2 (1.1**ab**)	0.09 (0.02**d**)	15.1 (0.08**e**)
Red Soil (pH=4.5)	30.4 (0.7**b**)	0.11 (0.06**d**)	16.2 (0.06**c**)
Red Soil (pH=5.5)	27.8 (0.6**c**)	0.13 (0.05**d**)	15.7 (0.14**d**)
Red Soil (pH=6.5)	30.7 (0.4**b**)	0.25 (0.06**c**)	16.4 (0.11**b**)
Red Soil (pH=7.5)	29.9 (1.2**b**)	0.57 (0.07**b**)	15.2 (0.11**e**)
Red Soil (pH=8.5)	32.6 (0.9**a**)	0.75 (0.02**a**)	16.6 (0.13**a**)

Values indicate the mean concentrations of Fe, Ca and SO_4^{2-} in soil samples. Values in brackets represent standard deviations (n = 12). Different letters in column (shown in Bold) indicate significant differences (*P*-value < 0.05, Duncan's multiple comparisons test) within the same soil type.

Supplementary Table 5. Summary of the reads of amplicon sequencing data and the assembly results of metagenomic sequencing data.

Soil pH	Black Soil		Red Soil						
	Amplicon sequencing		Amplicon sequencing		Metagenomic sequencing				
	Dilution level	No. of 16S reads	Dilution level	No. of 16S reads	Assembly rate (%)	total length (bp)	Average len(bp)	N50 Len(bp)	No. of unigenes
	Untreated Black Soil	22697	Untreated Red Soil	23874	66.47	1,982,255,184	758	838	38,531,429
4.5	Dil = 10^{-1}	17855	Dil = 10^{-1}	12331	63.04	3,241,428,864	710	748	37,351,896
	Dil = 10^{-4}	21055	Dil = 10^{-4}	11384	62.10	3,354,976,701	631	631	11,795,336
	Dil = 10^{-7}	20858	Dil = 10^{-7}	11166	64.80	3,227,262,438	672	671	2,359,067
	Dil = 10^{-10}	16703	Dil = 10^{-10}	13776	63.19	3,203,194,074	729	783	629,085
5.5	Dil = 10^{-1}	19005	Dil = 10^{-1}	12442	56.70	3,714,657,996	679	678	37,725,415
	Dil = 10^{-4}	22084	Dil = 10^{-4}	13015	56.22	3,727,109,277	662	645	11,913,289
	Dil = 10^{-7}	26696	Dil = 10^{-7}	12588	63.06	2,882,336,403	695	724	2,382,658
	Dil = 10^{-10}	19762	Dil = 10^{-10}	15634	57.30	2,737,577,775	585	554	635,375
6.5	Dil = 10^{-1}	18398	Dil = 10^{-1}	11469	58.74	2,898,502,167	663	646	38,102,669
	Dil = 10^{-4}	19209	Dil = 10^{-4}	12101	62.10	2,954,053,434	688	691	12,032,422
	Dil = 10^{-7}	28208	Dil = 10^{-7}	11970	57.92	3,279,099,201	681	687	2,406,484
	Dil = 10^{-10}	30306	Dil = 10^{-10}	12129	62.77	3,502,986,303	681	688	641,729
7.5	Dil = 10^{-1}	25539	Dil = 10^{-1}	11636	64.12	3,793,939,005	677	673	37,340,615
	Dil = 10^{-4}	25768	Dil = 10^{-4}	12118	62.07	3,557,786,829	690	693	11,791,773
	Dil = 10^{-7}	26905	Dil = 10^{-7}	11557	66.85	3,835,308,711	724	771	2,358,355
	Dil = 10^{-10}	28127	Dil = 10^{-10}	11610	63.02	3,189,474,909	689	715	628,895
8.5	Dil = 10^{-1}	23659	Dil = 10^{-1}	11020	66.58	3,256,577,337	682	704	36,593,803
	Dil = 10^{-4}	27587	Dil = 10^{-4}	11324	56.75	2,874,628,578	576	550	11,555,938
	Dil = 10^{-7}	27177	Dil = 10^{-7}	11759	57.30	2,777,649,639	588	560	2,311,188
	Dil = 10^{-10}	39488	Dil = 10^{-10}	13476	66.77	2,313,261,129	678	675	616,317

Supplementary Note 1 (R codes)

```
rm(list=ls())
data.set.name = 'test'
setwd("E:/NTI_files")

library(picante)

# read in a rarefied OTU table with all OTUs have abundance of 1 or greater.
otu =
as.data.frame(read.table(paste(data.set.name,"_rarefied_otu.txt",sep=""),header=T,ro
w.names=1));    # species as rows, samples as columns for otu table

# read in phylo and match
phylowb = read.tree(paste(data.set.name,"_phylo_tree.nwk",sep=""))
phylowb

match.phylowb.otu = match.phylowb.data(phylowb, t(otu))
str(match.phylowb.otu)

write.tree(match.phylowb.otu$phy,paste(data.set.name,"_matched_tree_to_rarified_ot
u.tre",sep=""))

phylowbMatch =
read.tree(paste(data.set.name,"_tree_matched_to_rarified_otu_table.tre",sep=""))
phyloMatch

match.phylowbMatch.otu = match.phylowbMatch.data(phylowbMatch, t(otu))
str(match.phylowbMatch.otu)

beta.mntd.weighted =
as.matrix(comdistnt(t(match.phylowbMatch.otu$data),cophenetic(match.phylowbMat
ch.otu$phy),abundance.weighted=T));
dim(beta.mntd.weighted);
beta.mntd.weighted[1:5,1:5];
write.csv(beta.mntd.weighted,'betaMNTD_weighted.csv',quote=F);

identical(colnames(match.phylowbMatch.otu$data),colnames(beta.mntd.weighted)); #
Just checking and should be TRUE
identical(colnames(match.phylowbMatch.otu$data),rownames(beta.mntd.weighted));
# Just checking and should be TRUE

# calculate randomized betaMNTD
```

```
beta.reps = 999; # number of randomizations

random.weighted.bMNTD.comp =
array(c(-999),dim=c(ncol(match.phylowbMatch.otu$data),ncol(match.phylowbMatch.otu$data),beta.reps));
dim(random.weighted.bMNTD.comp);

for (rep in 1:beta.reps) {

   random.weighted.bMNTD.comp[,,rep] =
as.matrix(comdistnt(t(match.phylowbMatch.otu$data),taxaShuffle(cophenetic(match.phylowbMatch.otu$phy)),abundance.weighted=T,exclude.conspecifics = F));

   print(c(date(),rep));

}

weighted.bNTI =
matrix(c(NA),nrow=ncol(match.phylowbMatch.otu$data),ncol=ncol(match.phylowbMatch.otu$data));

dim(weighted.bNTI);

for (columns in 1:(ncol(match.phylowbMatch.otu$data)-1)) {
   for (rows in (columns+1):ncol(match.phylowbMatch.otu$data)) {

      random.vals = random.weighted.bMNTD.comp[rows,columns,];
      weighted.bNTI[rows,columns] = (beta.mntd.weighted[rows,columns] -
mean(random.vals)) / sd(random.vals);
      rm("random.vals");

   };
};

rownames(weighted.bNTI) = colnames(match.phylowbMatch.otu$data);
colnames(weighted.bNTI) = colnames(match.phylowbMatch.otu$data);
weighted.bNTI;
write.csv(weighted.bNTI,"weighted_bNTI.csv",quote=F);
```
