# Supplementary material for: Diversity-triggered deterministic bacterial assembly constrains community functions
Source: Nat Commun. 2019 Aug 23;10:3833. doi: 10.1038/s41467-019-11787-5 (PMC6707308; doi:10.1038/s41467-019-11787-5)
Supplement: Supplementary file 3 — Reporting Summary [file 41467_2019_11787_MOESM3_ESM.pdf]

nature research

Corresponding author(s): Ruifu Zhang & Qirong Shen

Last updated by author(s): Apr 25, 2019

Reporting Summary

Nature Research wishes to improve the reproducibility of the work that we publish. This form provides structure for consistency and transparency in reporting. For further information on Nature Research policies, see Authors & Referees and the Editorial Policy Checklist.

Statistics

For all statistical analyses, confirm that the following items are present in the figure legend, table legend, main text, or Methods section.

n/a	Confirmed	
☐	☒	The exact sample size (*n*) for each experimental group/condition, given as a discrete number and unit of measurement
☐	☒	A statement on whether measurements were taken from distinct samples or whether the same sample was measured repeatedly
☐	☒	The statistical test(s) used AND whether they are one- or two-sided<br>*Only common tests should be described solely by name; describe more complex techniques in the Methods section.*
☒	☐	A description of all covariates tested
☐	☒	A description of any assumptions or corrections, such as tests of normality and adjustment for multiple comparisons
☐	☒	A full description of the statistical parameters including central tendency (e.g. means) or other basic estimates (e.g. regression coefficient) AND variation (e.g. standard deviation) or associated estimates of uncertainty (e.g. confidence intervals)
☐	☒	For null hypothesis testing, the test statistic (e.g. *F*, *t*, *r*) with confidence intervals, effect sizes, degrees of freedom and *P* value noted<br>*Give P values as exact values whenever suitable.*
☒	☐	For Bayesian analysis, information on the choice of priors and Markov chain Monte Carlo settings
☒	☐	For hierarchical and complex designs, identification of the appropriate level for tests and full reporting of outcomes
☐	☒	Estimates of effect sizes (e.g. Cohen's *d*, Pearson's *r*), indicating how they were calculated

Our web collection on statistics for biologists contains articles on many of the points above.

Software and code

Policy information about availability of computer code

Data collection	For the amplicon sequencing data analysis, we used the UPARSE pipeline (http://drive5.com/usearch/manual/uparse_pipeline.html). For the metagenomic sequencing data analysis, we used IDBA, MetaGeneMark, CD-HIT, SOAPaligner and BLAST. For statistical analysis we used FastUnifrac and R. All published open source softwares were described in methods section.
Data analysis	For the amplicon sequencing data analysis, we used the UPARSE pipeline (http://drive5.com/usearch/manual/uparse_pipeline.html). For the metagenomic sequencing data analysis, we used IDBA, MetaGeneMark, CD-HIT, SOAPaligner and BLAST. For statistical analysis we used FastUnifrac and R. All published open source softwares were described in methods section.

For manuscripts utilizing custom algorithms or software that are central to the research but not yet described in published literature, software must be made available to editors/reviewers. We strongly encourage code deposition in a community repository (e.g. GitHub). See the Nature Research guidelines for submitting code & software for further information.

Data

Policy information about availability of data

All manuscripts must include a data availability statement. This statement should provide the following information, where applicable:

- Accession codes, unique identifiers, or web links for publicly available datasets
- A list of figures that have associated raw data
- A description of any restrictions on data availability

The DNA sequences from all incubation samples are deposited in the NCBI Sequence Read Archive (SRA) database with accession numbers of SRR8857587, SRR8857588, SRR8857589, SRR8857590, SRR8857591 and SRR8840928. The authors declare that the data supporting the findings of this study are available within the paper and its Supplementary Information files.

Field-specific reporting

Please select the one below that is the best fit for your research. If you are not sure, read the appropriate sections before making your selection.

☒ Life sciences ☐ Behavioural & social sciences ☐ Ecological, evolutionary & environmental sciences

For a reference copy of the document with all sections, see nature.com/documents/nr-reporting-summary-flat.pdf

Life sciences study design

All studies must disclose on these points even when the disclosure is negative.

Sample size

We selected 2 soil types (black and red soils), 4 dilution levels (10-1, 10-4, 10-7 and 10-10) and 5 pH levels (4.5, 5.5, 6.5, 7.5 and 8.5) and established a total of 42 treatments (5 pH levels × 4 dilution levels × 2 soil types + 2 initial soils) with 6 biological replicates. For soil physic-chemical analysis, 6 samples from 6-replicated microcosms were used for each treatment when the incubation finished. For DNA extraction, 2 replicates of soil samples were collected from every microcosm. Therefore, we obtained 12 DNA replicates form every treatment. Sample size is reflected in Methods section.

Data exclusions

No data were excluded from the analyses.

Replication

We selected 2 soil types (black and red soils), 4 dilution levels (10-1, 10-4, 10-7 and 10-10) and 5 pH levels (4.5, 5.5, 6.5, 7.5 and 8.5) and established a total of 42 treatments (5 pH levels × 4 dilution levels × 2 soil types + 2 initial soils) with 6 biological replicates. For soil physic-chemical analysis, 6 samples from 6-replicated microcosms were used for each treatment when the incubation finished. For DNA extraction, 2 replicates of soil samples were collected from every microcosm. Therefore, we obtained 12 DNA replicates form every treatment. Sample size is reflected in Methods section.

Randomization

Each microcosm was constructed by placing 250 g of γ-irradiated sterilized soil into a 500-ml bottle. Sterile distilled water was added to maintain a constant moisture level of 45% of field capacity, and the microcosms were pre-incubated at 20 °C in the dark for 4 weeks before being tested for sterility on agar plates. All microcosms were randomly chosen for pH manipulation and inoculation.

Blinding

Not applicable. All the incubation conditions are established and enclosed.

Reporting for specific materials, systems and methods

We require information from authors about some types of materials, experimental systems and methods used in many studies. Here, indicate whether each material, system or method listed is relevant to your study. If you are not sure if a list item applies to your research, read the appropriate section before selecting a response.

Materials & experimental systems

n/a	Involved in the study	
☒	☐	Antibodies
☒	☐	Eukaryotic cell lines
☒	☐	Palaeontology
☒	☐	Animals and other organisms
☒	☐	Human research participants
☒	☐	Clinical data

Methods

n/a	Involved in the study	
☒	☐	ChIP-seq
☒	☐	Flow cytometry
☒	☐	MRI-based neuroimaging
